# Paris

**Published:** 1894-06-23

**Authors:** 


### Continental Notes from a Holiday Correspondent.

PARIS.

Of all great centres of medical and scientific work on the Continent Paris is the most easily reached, and best repays a short visit. Foreign medical men are made most welcome at the hospitals and clinics, and so much is going on that one can fill one's mornings with visits to the various branches of the medical school. If our object is recreation as well as study, the afternoons can be devoted to the various sights of the city, or excursions in its neighbourhood. Indeed, considering its attractions it is wonderful how few English medical men visit Paris—at any rate in a professional capacity. Ignorance of the language need be no barrier, for in almost every clinic men will be found who speak English, often most fluently. Living is very economical, if one seeks for economy. In the Latin Quarter, where most of the medical schools are situated, one can get bed and breakfast for 4 francs at numerous small hotels, and at the Duval Restaurants, which are to be found all over the city, luncheon and dinner can be had for 4 francs more, if one chooses the more economical dishes. Allowing something for sundry small expenses, one can easily live for 10 francs, or 8s. a day, and not be stinted in any way as regards necessaries. Of course, this does not include anything for amusements. As regards the time of year for a visit, July and August should be avoided; but late in May and early in June Paris is at its best, and the weather is generally delightful. Late in September is also good. At these times the hospitals are in full swing, while in the hotter weather many men will be away.

The hospitals are very numerous, and the best plan for a medical visitor is to buy the little guide, published at the office of *La Semaine Medicale*, and learn from it before he starts just where he should go to see the work he is most interested in. Besides the large public hospitals, there are a number of private clinics, held by men who have no hospital appointments. These must be inquired for among medical men working in Paris, who can give particulars. The chief general hospital is the Hôtel Dieu, and in it a number of Americans will always be found who are most willing to assist the English stranger. These private clinics should on no account be missed, for some of the best work is seen at them. In ophthalmic work, for instance, such men as Landolt and de Wecker are only to be seen at their own clinics, where visitors are made most welcome.

The Hôtel Dieu, the chief general hospital, is a splendid building. It is situated on the north side of the square of which the Cathedral of Notre Dame forms the east side. It was erected in 1877, close to the site of an older hospital, which was founded by Clovis II. in 660. It cost almost two million sterling, but the money might have been better spent elsewhere, for about half of it was paid for the site, and this site by no means a good one from a sanitary point of view, for it lies low on an island in the Seine.

The hospital contains 559 beds, and is splendidly equipped for teaching purposes as well as for the treatment of medical and surgical ailments. It is built in six pavilions—two parallel rows of three. The floors are polished and the walls glazed to secure the utmost cleanliness, and in all departments the most rigorous and elaborate aseptic arrangements prevail. The staff consists of six physicians and three surgeons, but a great deal of the work is done by assistants.

Suppose we go to the ophthalmic clinic, for instance. We find M. Panas seated by a table in a room about 30 feet square, with high ceiling, and well lighted on one side. Round him are seated some fifteen or twenty students and graduates, many of them Americans, Germans, and other foreigners—and always some Greeks, his fellow countrymen. Five or six assistants in long white coats are bustling about. The chief assistant sees the patients in another room and sends in selected cases to M. Panas, writing the diagnosis on a slip of paper to save time. M. Panas reads this and examines the patient, spending a long time on each. After about an hour spent this way he goes to the theatre and lectures for another hour, usually on some case he has seen that morning. Then come operations which may go on till two o'clock. The work strikes one as very carefully done, and the facilities for learning are greater than is the case with us.

As an example of the private clinic we may take M. de Wecker's, at the Rue du Cherche Midi, 55. The house is a fine old one, evidently once an aristocratic dwelling-house, as is seen by the carved doors and the painted panels over them. The upper floors are used as a private hospital, and part of the ground floor as a private consulting-room, while the other part is the public clinic. Here patients are seen on Mondays, Wednesdays, and Fridays at half-past four, and after they have been seen the operations are performed. M. de Wecker is a most good-natured old gentleman, chatting away to everyone all round, speaking both French and English with a strong German accent. Needless to say, his fame attracts many visitors, and no one could be more polite to them than he.

